# Clinical Outcomes of Ileostomy Closure before Adjuvant Chemotherapy after Rectal Cancer Surgery: An Observational Study from a Chinese Center

**DOI:** 10.1155/2021/5592721

**Published:** 2021-07-13

**Authors:** Zhen Sun, Yufeng Zhao, Lu Liu, Jichao Qin

**Affiliations:** ^1^Department of Gastrointestinal Surgery, Tongji Hospital, Tongji Medical College in Huazhong University of Science and Technology, Wuhan, Hubei, China; ^2^Tongji Cancer Research Institute, Tongji Hospital, Tongji Medical College in Huazhong University of Science and Technology, Wuhan, Hubei, China

## Abstract

**Background:**

The optimal timing of temporary ileostomy closure with respect to the time of adjuvant chemotherapy following sphincter-saving surgery for rectal cancer remains unclear. The aim of this study is to investigate the clinical and oncological outcomes of ileostomy closure before, during, and after adjuvant chemotherapy following curative rectal cancer resection.

**Methods:**

Patients diagnosed with rectal adenocarcinoma who underwent low anterior resection and temporary loop ileostomy during May 2015 and September 2019 were retrospectively evaluated. Patients undergoing ileostomy closure before adjuvant chemotherapy (Group I) were compared to patients undergoing closure during (Group II) and after (Group III) adjuvant chemotherapy.

**Results:**

A total of 225 patients were evaluated for eligibility, and 132 were finally selected and divided into 3 groups (24 in Group I, 53 in Group II, and 55 in Group III). No significant differences were observed in operative time, postoperative hospital stay, postoperative complications, total adjuvant chemotherapy cycles, and low anterior resection syndrome scores among the three groups. There was no significant difference in disease-free survival (*p* = 0.834) and overall survival (*p* = 0.462) between the three groups.

**Conclusion:**

Temporary ileostomy closure before adjuvant chemotherapy following curative rectal cancer resection can achieve a clinical and oncological safety level equal to stoma closure during or after chemotherapy in selected patients.

## 1. Introduction

A temporary diverting ileostomy is frequently performed on patients who have had rectal cancer surgery to protect anastomosis, particularly for rectal cancer of the middle and lower third [1, 2]. Previous studies have demonstrated that patients with a temporary diverting ileostomy were at lower risk to suffer from anastomotic leakage, peritonitis, and their associated morbidity and mortality than those without it [3, 4].

There are some agreements among surgeons that ileostomy closure should not be performed earlier than 60-90 days [5, 6]. However, a diverting stoma has various adverse effects including stoma-related morbidities, physical stress, and psychological handicap, which affect the patients' quality of life [7, 8]. Therefore, patients suffering from considerable pressure are eager to close the diverting stoma after primary surgery; however, there are no strict recommendations for the optimal timing of ileostomy closure. Recently, several studies have addressed the safety and feasibility of early ileostomy closure (within 2 weeks after primary surgery) and reported promising results [9–11]. After rectal cancer surgery, the outcomes of early versus late closure of loop ileostomy have been studied; however, the outcomes remain controversial with respect to adjuvant chemotherapy. Studies showed that temporary ileostomy closure before [12, 13] or during [14–17] adjuvant chemotherapy following rectal cancer surgery had similar outcomes to the closure of ileostomy after adjuvant chemotherapy. On the contrary, other studies showed that a shorter interval between primary surgery and ileostomy closure may negatively affect the completeness of chemotherapy resulting from stoma-related complications such as anastomotic leakage or incomplete anorectal function [18–20]. In addition, stoma closure before or during adjuvant chemotherapy may induce the delay or interruption of chemotherapy, which may alter the effects of chemotherapy [21, 22].

Considering the controversies regarding the optimal timing of temporary ileostomy closure with respect to the time of delivering adjuvant chemotherapy, our goal was to investigate the outcomes of ileostomy closure before, during, and after adjuvant chemotherapy following curative rectal cancer surgery.

## 2. Method

### 2.1. Patients and Interventions

Patient data was extracted from a prospectively collected colorectal cancer database retrospectively. Patients diagnosed with rectal adenocarcinoma who received low anterior resection with total mesorectal excision (TME) and temporary loop ileostomy closure between May 2015 and September 2019 were included. Informed consent had been obtained from individual patients, and the present study had been approved by the institutional review board of the hospital.

Exclusion criteria included patients with stage IV rectal cancer and postoperative radiotherapy or patients who suffered complications of anastomosis from the index surgery. Patients who did not undergo adjuvant chemotherapy were also excluded.

We divided our patients into three groups: Group I, Group II, and Group III, who underwent ileostomy closure before, during, and after chemotherapy, respectively. The demographics and clinical characteristics were compared between the groups.

The anastomosis site was assessed with contrast enema, abdominopelvic computed tomography, or/and colorectal endoscopy to ensure the safety of the ileostomy closure. The ileostomy closure technique included handsewn or stapled ileo-ileal anastomosis, which was left to the discretion of the surgeon. Eight different gastrointestinal surgeons working in the same institution performed all procedures.

### 2.2. Outcomes

The compared parameters included details of surgical procedures, demographics, clinical characteristics, stoma closure, length of hospital stay, information of adjuvant chemotherapy, complications related to the stoma formation, prevalence of low anterior resection syndrome (LARS), disease recurrence, and survival.

The stoma formation-related complications were extracted retrospectively by inspection of patient charts filled in by stoma care therapists. High volume output was defined as the combination of stoma content output of more than 1000 ml and electrolyte disturbance. Skin irritation included parastomal infection, rash, pain, or ecchymoma. Stoma closure-related complications were assessed within 30 days after ileostomy closure.

The overall survival (OS) was presented by the interval between index surgery and death, while the disease-free survival (DFS) was defined by the interval between index surgery and the date of the first recurrence (local and/or distant). Data on patients who were disease-free were censored from the date of the last follow-up until May 2020. Meanwhile, a LARS score questionnaire [23] was completed by the follow-up telephone calls to the surviving participants. The associated response categories were based on the frequency of symptom occurrence or number of bowel motions. A LARS score of 0–20 was interpreted as no LARS; 21–29, minor LARS; and 30–42, major LARS [23].

### 2.3. Statistical Analysis

Continuous parameters were presented as the mean and standard deviation and were further analyzed using one-way analysis of variance. The categorical parameters were described using percentages, the chi-squared test, or the Fisher exact test. The survival curve between groups regarding OS and DFS was calculated by Kaplan-Meier analysis and compared with the log-rank test. SPSS statistical software version 19.0 (SPSS Inc., Chicago, IL, USA) for Windows and GraphPad Prism 5 (GraphPad Software Inc., San Diego, CA) were applied for all data analyses. *p* < 0.05 was considered statistically significant.

## 3. Results

**Figure 1:**
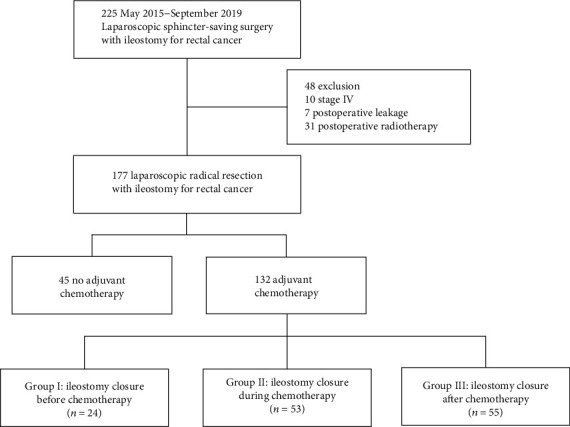
Flowchart of patients included in the study.

A total of 225 patients were evaluated for eligibility, and 132 were included and divided into three groups in the present study: Group I consisted of 24 patients and underwent ileostomy closure before chemotherapy; Group II consisted of 53 patients and underwent it during chemotherapy; and Group III consisted of 55 patients and underwent it after chemotherapy (Figure 1). The demographics and clinicopathologic characteristics among the three groups were comparable (Table 1). The preoperative BMI (body mass index) of patients in Group III was higher, and the difference nevertheless was not statistically significant.

The mean duration of the diverting stoma was 25.9 ± 5.3 days in Group I, 119.3 ± 47.5 days in Group II, and 202.3 ± 93.8 days in Group III (*p* < 0.0001) (Table 2). Intraoperative features such as the operative time and anastomosis method were comparable among the three groups. Notes about unexpected difficulties including mobilization of ileum adhesion or mobilization of ileostomy from the abdominal were described more often in Group I than the other two groups (29.2% in Group I vs. 11.3% in Group II and 9.1% in Group III, *p* = 0.047). No significant difference was observed in the interval for resuming diet, passing of gas, postoperative 30-day mortality, reoperation rate, and total adjuvant chemotherapy. The postoperative hospital stay was longer, but not statistically significant, in Group I than the others (8.6 ± 2.2 days in Group I vs. 7.5 ± 2.5 days in Group II and 7.5 ± 2.1 days in Group III, *p* = 0.121). The mean interval between index surgery and first adjuvant chemotherapy was 51.5 ± 7.0 days in Group I, 33.8 ± 12.4 days in Group II, and 30.2 ± 9.7 days in Group III (*p* < 0.0001).

Overall, no significant difference was detected among the three groups regarding the incidence of stoma formation-related complications. Six (10.9%) patients in Group III had skin irritation after stoma formation, significantly higher than Group I (4.2%) and Group II (0.0%) (*p* = 0.039) (Table 3). One patient from Group I underwent stoma closure during the index surgery admission period for stoma prolapse and skin irritation. None of the patients required emergency surgery due to high volume output or postoperative ileus. Overall, the incidence of stoma closure-related complications was 20.8% in Group I, 13.2% in Group II, and 12.7% in Group III, which was not statistically different. No fistula and anastomotic leakage were observed among all three of the groups. The wound infection rate was higher but not significant in Group I (12.5%) than in Group II (7.5%) and Group III (9.1%) (*p* = 0.783). Two patients in Group III and one patient in Group II underwent reoperation due to incisional hernia on the stoma closure site 1 month after ileostomy closure. In addition, one patient in Group I underwent reoperation for postoperative ileus who did not succeed in conservative treatment.

**Figure 2:**
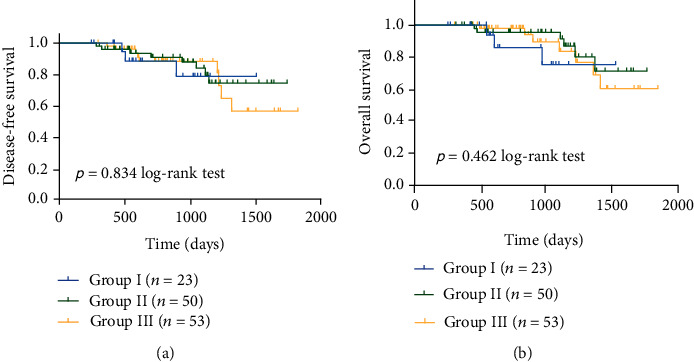
Kaplan-Meier curves of survival between the three groups: (a) disease-free survival and (b) overall survival.

126 of the original 132 participants were available in the survival analysis. The average follow-up period in Group I was 731 ± 332 days; in Group II, 977 ± 399 days; and in Group III, 897 ± 389 days. No differences were found in OS and DFS among the three groups (*p* = 0.462 for OS and *p* = 0.834 for DFS) (Figure 2).

The LARS score questionnaire was completed by all 106 surviving patients among the 126 participants for survival analysis. The demographics and clinicopathologic characteristics between the three groups among these participants were comparable. Overall, the incidence of LARS was 50.0% in Group I, 54.7% in Group II, and 47.7% in Group III. The median LARS scores for Groups I-III were 17 (interquartile range 6-29), 24 (14-32), and 20 (8-31), respectively (*p* = 0.282). No significant differences were noticed regarding incontinence to feces and flatus, increased stool frequency, clustering, and urgency among the three groups (Table 4).

## 4. Discussion

In this study, we compared the postoperative morbidity and mortality of ileostomy closure before, during, and after adjuvant chemotherapy after the curative rectal cancer resection and tried to find out whether survival rates and recurrence are associated with the time until closure. We found that in selected patients, temporary ileostomy closure before adjuvant chemotherapy could achieve a clinical safety level equal to stoma closure during or after chemotherapy in terms of postoperative complication, LARS, and oncological prognosis.

There is uncertainty about the timing of the closure of ileostomy since most patients with rectal cancer are likely to receive postoperative adjuvant chemotherapy. The interval between creation and closure of the temporary stoma is often delayed in rectal cancer patients who had received adjuvant chemotherapy [24–26]. These patients suffered more stoma-related complications before closure which negatively impact their quality of life [7, 8, 27]. Whether the closure of a temporary stoma can be done before or during adjuvant chemotherapy instead of after needs clear guidelines. Thalheimer et al. [12] found that fewer complications happened in the cases of ileostomy closure performed before (12.5%) the start of adjuvant chemotherapy or radiochemotherapy, rather than during (42.9%) or after (21.2%). They speculated that the higher complication rates in the latter two groups might be because of the patients' compromised general physical condition during and after the adjuvant therapy lasting up to 6 months, such as decreased wound healing capacity. Furthermore, Lordan et al. [24] pointed out that it would be feasible to close the temporary stoma before starting adjuvant therapy because most times the postoperative therapy is not initiated in 2-3 weeks after the anterior resection. This would decrease the incidence of stoma-related complications and avoid a long delay in closure after adjuvant therapy. Recently, Kłęk et al. [13] reported that ileostomy closure performed in advance of adjuvant chemotherapy was safe and should be considered part of the enhanced recovery after surgery (ERAS) protocol. In our study, we found that stoma formation-related complication rates in Group III were higher than those in Group I or Group II. In particular, the patients in Group III suffered more skin irritation than those in the other two groups. Therefore, patients suffering from considerable pressure tend to choose to close the diverting stoma at an early stage.

In the present study, pairwise comparisons among all groups indicated that there were no significant differences between them in the stoma closure-related complications, reoperation rate, and mortality. Some notes about unexpected difficulties were described more often in Group I than the other two groups. This could be because the collagen synthesis and the inflammatory process remain active until 4 weeks after index surgery [28]. In addition, preoperative radiotherapy induced inflammation and fibrosis needs time to regenerate and absorb [29] which could also explain a higher peristomal adhesion in Group I patients. Studies focusing on the timing of ileostomy closure after the neoadjuvant chemoradiotherapy (CRT) or total neoadjuvant treatment (TNT) in locally advanced rectal cancer need further investigation. In spite of this, the time of operation, passing of gas, fully oral nutrition, and postoperative hospital stay were similar among the three groups indicating that the peristomal adhesion is no longer an obstacle with the enhanced surgical technique.

Wound infection is a relatively common stoma closure-related complication. In the present study, we defined wound infection as the redness or tenderness of the surgical wound with the discharge of pus [30]. We found that wound infection after stoma closure was higher but not significant in Group I (12.5%) than in Group II (7.5%) and Group III (9.1%) (*p* = 0.783). One meta-analysis reported that the wound infection rate was 15.5% in patients who reversed their stoma within 2 weeks and 5.3% in patients who reversed their stoma at least 8 weeks after rectal surgery [11]. These results could be explained by the reduction of the recovery or immunity of patients in the immediate postoperative period, which leads the host susceptible to infectious complications through diverse cytokine activities [9, 31]. In addition, preoperative nutritional status is also one of the major risk factors for wound infection in patients undergoing abdominal surgery [30]. In the present study, the BMI and preoperative albumin level in Group I were lower than those in the other two groups which also might explain the higher frequency of wound infection in Group I. Therefore, to improve clinical outcomes, clinicians should reduce wound infection through preoperative nutrition support, accounting for the purse-string skin closure technique [14] in early stoma closure patients.

It is recommended to begin adjuvant chemotherapy up to 8 weeks from the date of colorectal cancer surgery [21]. Recently, surgeons [9–11] have claimed that early ileostomy closure within 2 weeks is safe, providing the patients with enough time to recover and undergo adjuvant therapy [13]. In the present study, the mean interval between index surgery and first adjuvant chemotherapy was 51.5 ± 7.0 days in Group I, suggesting that patients in the early ileostomy closure group did not significantly exceed recommended duration between radical surgery and the start of chemotherapy. In addition, our results showed that no significant differences were found in OS and DFS among the three groups, thus indicating that early stoma closure before adjuvant chemotherapy did not affect oncological outcomes [15, 17]. However, some surgeons and oncologists are reluctant to stoma closure before adjuvant chemotherapy because it is associated with a 17% postoperative morbidity rate [32], which may affect the initiation of adjuvant chemotherapy. Although the clinical and oncological outcomes are comparable among groups in the present study, the mean duration of the diverting stoma is 25.9 ± 5.3 days in Group I which is still relatively longer than other studies [9–11]. In order to start the adjuvant treatment in time, the ileostomy closure time needs to be shortened. In the future, our center will initiate a randomized controlled study in which the safety and feasibility of ileostomy closure will be evaluated 14 days after index surgery. In addition, a method to reduce postoperative morbidity should also be considered to shorten the interval of the ileostomy closure and the first chemotherapy. Two patients in Group I wanted more recovery time because of the postoperative ileus. The delay of adjuvant chemotherapy in these patients should be taken into account, and excessive delay should be prevented in adjuvant treatment. Among the 3 patients that required treatment for an ileostomy closure wound, the adjuvant chemotherapy was about 1 week delayed. In such cases, chemotherapy might not be delayed if the preoperative nutrition support or purse-string skin closure technique was taken into account. In addition, patients who underwent postoperative radiotherapy were not included in the present study because such treatment strategy may increase the risk of anastomotic leakage [33] and prolong the stoma closure time. In the future, ileostomy closure before chemotherapy should only be proposed for carefully selected patients without any signs of anastomotic leakage and uneventful postoperative outcomes. Therefore, in order to improve this selection process, another study to determine the risk factors for complications after loop ileostomy closure following sphincter-saving surgery with respect to adjuvant chemotherapy is ongoing in our center.

LARS is frequently reported in patients with rectal cancer who received TME with low colorectal anastomosis. The disordered defecatory function during LARS, such as increased stool frequency, clustering, urgency, and incontinence to feces and flatus after sphincter-saving procedures, negatively affects the patient's quality of life [34]. Opinions on the effect of a diverting stoma on the incidence and severity of anorectal functional alterations after anterior resection are controversial. In addition, the relationship between the timing of ileostomy closure and LARS is rarely reported. The pathophysiology leads us to believe that the disuse colitis and delayed restoration of bowel continuity may result in alterations in colonic nutrition, causing inflammation, changes in the bacterial flora and irreversible colon, and rectal atrophy of motility or sensory elements [23, 35, 36]. Recently, a prospective randomized controlled trial found earlier closure of ileostomy after anterior resection had a better LARS score than the later closure group, although the difference was not statistically significant. Several noncontrolled studies have also shown an association between the use of a diverting stoma and LARS in univariate analysis, indicating that the timely restoration of bowel continuity might avoid the irreversible colon and rectal atrophy and reduce the incidence of LARS [37–39]. However, confounding factors such as age, gender, tumor location, and perioperative radiotherapy need to be considered when interpreting these results. Notably, some other studies have found no difference in anorectal function between patients with and without a temporary defunctioning stoma [40, 41]. In the present study, the median LARS score in Group I was better than that in the other two groups, indicating that the early ileostomy closure might improve functional outcomes in these patients. Alternatively, a temporary loop ileostomy closure before adjuvant chemotherapy is comparable with the closure of ileostomy during or after adjuvant chemotherapy regarding the anorectal function. The controversial results mentioned above suggest that it is essential to apply powered prospective randomized studies to evaluate definitively whether early closure of an ileostomy could decrease the development of LARS.

Our study had some limitations, namely, because it is retrospective. Next, a lack of randomization to different groups creates bias. Surgeon preference and patient desire affect this selection. While some surgeons in this study were used to perform ileostomy closure after completion of chemotherapy, other surgeons choose to close an ileostomy during or before chemotherapy. Patient status such as systemic illness, variables during index surgery, or stoma formation-related complications may also play an important role in affecting this selection. In addition, a surgeon's decision is compromised due to a patient's strong desire to close the stoma. Nevertheless, our study has its advantages due to the sparse literature available reporting the safety of ileostomy closure before adjuvant chemotherapy regarding postoperative complications, which provides some hints for clinicians to make better clinical decisions on the optimal timing of ileostomy closure with respect to adjuvant chemotherapy. Furthermore, this is the first study comparing the effect of ileostomy closure before and during or after adjuvant chemotherapy on oncologic outcomes.

In conclusion, our findings suggest that after colorectal cancer resection, performing temporary loop ileostomy closure before adjuvant chemotherapy has comparable effects with the closure of ileostomy during or after adjuvant chemotherapy in terms of postoperative complication, LARS, and oncological prognosis in selected patients. A well-planned larger-scale, randomized, controlled trial with a long follow-up should be performed to accurately define which individuals stand to benefit from early closure of ileostomy before adjuvant chemotherapy and to assess this strategy with regard to the quality of life and compliance of adjuvant chemotherapy.

## Figures and Tables

**Table 1:** Demographics and clinicopathologic characteristics of the study groups.

	Group I (*n* = 24)	Group II (*n* = 53)	Group III (*n* = 55)	*p* value
Sex				
Male	16 (66.7%)	41 (77.4%)	32 (58.2%)	0.104
Female	8 (33.3%)	12 (22.6%)	23 (41.18%)	
Age (years)	57.2 ± 9.7	56.3 ± 10.0	53.7 ± 11.2	0.304
BMI^∗^ (kg/m^2^)	21.1 ± 2.4	22.3 ± 3.4	22.8 ± 2.7	0.073
Comorbidity				
Cardiovascular diseases	2 (8.3%)	8 (15.1%)	12 (21.8%)	0.310
Diabetes	0 (0.0%)	6 (4.5%)	3 (2.3%)	0.165
Pulmonary diseases	1 (4.2%)	1 (1.9%)	1 (1.8%)	0.789
Renal diseases	0 (0.0%)	1 (1.9%)	2 (3.6%)	0.590
ASA class				0.961
I	3 (12.5%)	5 (9.4%)	4 (7.3%)	
II	19 (79.2%)	44 (83.0%)	47 (85.5%)	
III	2 (8.3%)	4 (7.5%)	4 (7.3%)	
Type of surgery				0.994
Laparoscopic	23 (95.8%)	51 (96.2%)	53 (96.4%)	
Conversion to open	1 (4.2%)	2 (3.8%)	2 (3.6%)	
cTNM stage				0.706
I	1 (4.2%)	5 (5.7%)	18 (7.3%)	
II	5 (20.8%)	17 (32.1%)	33 (21.8%)	
III	18 (75.0%)	33 (62.3%)	39 (70.9%)	
ypTNM stage^∗∗^				0.471
0	1 (4.2%)	0 (0.0%)	1 (1.8%)	
I	2 (8.3%)	4 (7.5%)	9 (16.4%)	
II	12 (50.0%)	25 (47.2%)	28 (50.9%)	
III	9 (37.5%)	24 (45.3%)	17 (30.9%)	
Neoadjuvant CRT	10 (41.7%)	19 (35.8%)	25 (45.5%)	0.595
Tumor location^∗∗∗^ (cm)	8.2 ± 3.8	7.5 ± 3.6	8.0 ± 3.7	0.698
3-5	9 (37.5%)	22 (41.5%)	16 (29.1%)	0.597
6-10	9 (37.5%)	23 (43.4%)	27 (49.1%)	
11-15	6 (25.0%)	8 (15.1%)	12 (21.8%)	

Values are presented as the mean ± standard deviation or number of patients (%). ^∗^Measured before ileostomy closure. ^∗∗^Pathological stage according to UICC. ^∗∗∗^Tumor lower border from the anal verge. BMI: body mass index; ASA: American Society of Anesthesiologists; UICC: Union Internationale Contre le Cancer; CRT: chemoradiotherapy.

**Table 2:** Details of loop ileostomy closure.

	Group I (*n* = 24)	Group II (*n* = 53)	Group III (*n* = 55)	*p* value
Interval to ileostomy closure (day)	25.9 ± 5.3	119.3 ± 47.5	202.3 ± 93.8	<0.001
Operative time (min)	139.8 ± 30.8	132.2 ± 26.9	130.3 ± 25.0	0.344
Anastomosis (ileo-ileal anastomosis)				0.613
Handsewn	14 (58.3%)	37 (69.8%)	36 (65.5%)	
Stapled	10 (41.7%)	16 (30.2%)	19 (34.5%)	
Noted unexpected difficulties during surgery	7 (29.2%)	6 (11.3%)	5 (9.1%)	0.047
Time until passing of gas (day)	3.1 ± 0.7	3.0 ± 0.6	2.8 ± 0.6	0.069
Time until fully oral nutrition (day)	2.8 ± 1.1	2.6 ± 0.7	2.4 ± 0.7	0.108
Stoma formation-related complications	3 (13.0%)	9 (17.0%)	11 (20.0%)	0.755
Stoma closure-related complication	5 (20.8%)	7 (13.2%)	7 (12.7%)	0.609
Postoperative 30-day mortality	0 (0%)	0 (0%)	0 (0%)	NA
Reoperation	1 (4.2%)	1 (1.9%)	2 (3.6%)	0.815
Hospital stay after closure (day)	8.6 ± 2.2	7.5 ± 2.5	7.5 ± 2.1	0.121
Total adjuvant chemotherapy (cycle)	5.2 ± 2.0	5.7 ± 1.4	4.9 ± 2.0	0.060
Interval between index surgery and 1^st^ chemotherapy (day)	51.5 ± 7.0	33.8 ± 12.4	30.2 ± 9.7	<0.001

Values are presented as the mean ± standard deviation or number of patients (%). NA: not available.

**Table 3:** Stoma-related complications.

	Group I (*n* = 24)	Group II (*n* = 53)	Group III (*n* = 55)	*p* value
Stoma formation-related complications	3 (13.0%)	9 (17.0%)	11 (20.0%)	0.755
High volume output	1 (4.2%)	5 (9.4%)	5 (9.1%)	0.715
Adhesive ileus	1 (4.2%)	4 (7.5%)	3 (5.5%)	0.822
Parastomal hernia	1 (4.2%)	0 (0%)	2 (3.6%)	0.353
Prolapse	1 (4.2%)	0 (0%)	0 (0%)	0.104
Skin irritation	1 (4.2%)	0 (0%)	6 (10.9%)	0.039
Stoma closure-related complications	5 (20.8%)	7 (13.2%)	7 (12.7%)	0.609
Wound infection	3 (12.5%)	4 (7.5%)	5 (9.1%)	0.783
Adhesive ileus	2 (8.3%)	2 (3.8%)	1 (1.8%)	0.378
Fistula/anastomotic leakage	0 (0%)	0 (0%)	0 (0%)	NA
Incisional hernia	0 (0%)	1 (1.9%)	2 (3.6%)	0.590

Values are presented as the number of patients (%). NA: not available.

**Table 4:** Details of LARS at follow-up 12 months after index surgery.

	Group I (*n* = 20)	Group II (*n* = 42)	Group III (*n* = 44)	*p* value
12-month median LARS scores (IQR)	17 (6-29)	24 (14-32)	20 (8-31)	0.282
Major LARS, *n* (%)	5 (25.0%)	14 (33.3%)	14 (31.8%)	0.796
Minor LARS, *n* (%)	5 (25.0%)	9 (21.4%)	7 (15.9%)	0.660
Incontinence to feces, *n* (%)	8 (40.0%)	19 (45.2%)	15 (31.4%)	0.572
Flatus, *n* (%)	1 (5.0%)	3 (7.1%)	5 (11.4%)	0.644
Increased stool frequency, *n* (%)	10 (50.0%)	28 (66.7%)	21 (47.7%)	0.179
Clustering, *n* (%)	14 (70.0%)	36 (85.7%)	30 (68.2%)	0.138
Urgency, *n* (%)	10 (50.0%)	27 (64.3%)	26 (59.1%)	0.563

Values are presented as the number of patients (%). LARS: low anterior resection syndrome; IQR: interquartile range.

## Data Availability

Data are available on request from the authors.
